# Identification of Somatic Mutation-Driven Immune Cells by Integrating Genomic and Transcriptome Data

**DOI:** 10.3389/fcell.2021.715275

**Published:** 2021-07-21

**Authors:** Ying Jiang, Baotong Zheng, Yang Yang, Xiangmei Li, Junwei Han

**Affiliations:** ^1^College of Basic Medical Science, Heilongjiang University of Chinese Medicine, Harbin, China; ^2^College of Bioinformatics Science and Technology, Harbin Medical University, Harbin, China

**Keywords:** multi-omics data, somatic mutations, tumor-infiltrating immune cells, tumor microenvironment, gene expression

## Abstract

Tumor somatic mutations in protein-coding regions may generate neoantigens which may trigger antitumor immune cell response. Increasing evidence supports that immune cell response may profoundly influence tumor progression. However, there are no calculated tools to systematically identify immune cells driven by specific somatic mutations. It is urgent to develop a calculated method to comprehensively detect tumor-infiltrating immune cells driven by the specific somatic mutations in cancer. We developed a novel software package (SMDIC) that enables the automated identification of somatic mutation-driven immune cell. SMDIC provides a novel pipeline to discover mutation-specific immune cells by integrating genomic and transcriptome data. The operation modes include inference of the relative abundance matrix of tumor-infiltrating immune cells, detection of differential abundance immune cells with respect to the gene mutation status, conversion of the abundance matrix of significantly dysregulated cells into two binary matrices (one for upregulated and one for downregulated cells), identification of somatic mutation-driven immune cells by comparing the gene mutation status with each immune cell in the binary matrices across all samples, and visualization of immune cell abundance of samples in different mutation status for each gene. SMDIC provides a user-friendly tool to identify somatic mutation-specific immune cell response. SMDIC may contribute to understand the mechanisms underlying anticancer immune response and find targets for cancer immunotherapy. The SMDIC was implemented as an R-based tool which was freely available from the CRAN website https://CRAN.R-project.org/package=SMDIC.

## Introduction

Tumor generally harbors somatic mutations, some of which in protein-coding regions may generate neoantigens which may trigger antitumor immune cell response in the tumor microenvironment (TME) (Yang et al., 2019). The mutation-reactive immune cells are usually found infiltrating into solid tumors (Robbins et al., 2013), and they profoundly influence tumor initiation, progression, metastasis, and treatment response (Chen and Mellman, 2017). For example, Tran et al. (2014) identify erbb2 interacting protein (ERBB2IP) mutation-specific CD4^+^ T (TH1) cells in a patient with metastatic cholangiocarcinoma and demonstrate that the ERBB2IP mutated-specific TH1 cell response can be used to mediate regression of metastatic epithelial cancer. Therefore, identifying mutation-specific immune cell response will illustrate the mechanisms underlying the anticancer immune response and might help to find targets for cancer immunotherapy (Yang et al., 2019). However, there are very few calculated tools that could comprehensively identify which immune cells are triggered by which mutations. Thus, it is urgent to develop a calculated method to comprehensively detect tumor-infiltrating immune cells driven by the specific somatic mutations in cancer. Recently, a number of computational approaches have been developed for quantifying tumor-infiltrating immune cells using tumor bulk gene expression data (Newman et al., 2015; Senbabaoglu et al., 2016; Aran et al., 2017). This will help to examine the correlation among somatic mutations and immune cells by using high-throughput sequencing data, for example whole-exome sequencing and RNA-seq.

Here, we developed a novel software package, SMDIC, to identify immune cells driven by specific somatic mutations by integrating genomic and transcriptome data, which is available at https://CRAN.R-project.org/package=SMDIC. SMDIC also provides visualization of the relative abundance of identified immune cells in the gene mutation status using heat maps, as well as the waterfall plot of mutation genes correlated with immune cells and mutually exclusive and co-occurring plot. We used the Genomic Data Commons (GDC) TCGA breast cancer somatic mutation and expression data to show the applications of the package and the visualization of the results. The results showed that SMDIC could effectively identify immune cells which may be driven by the specific somatic mutation genes, with strong robustness.

## Materials and Methods

**FIGURE 1:**
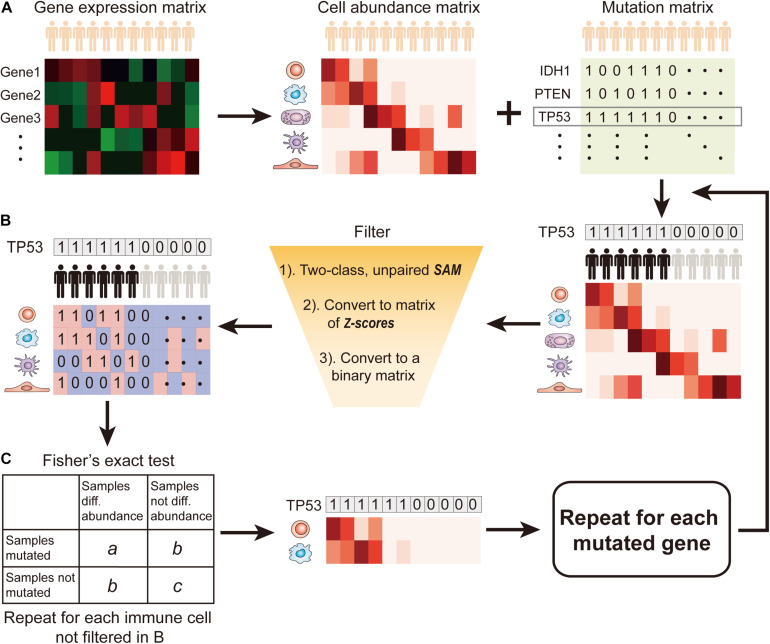
Flow diagram of SMDIC. **(A)** Inference of the relative abundance matrix of immune cells; **(B)** Detection of differential abundance immune cells with respect to the gene mutation status; **(C)** Identification of significant immune cells associated with somatic mutation.

SMDIC has three main functions (Figure 1): (a) inferring the relative abundance matrix of tumor-infiltrating immune cells, (b) detecting differential abundance immune cells with respect to a particular gene mutation status and converting the abundance matrix of significantly dysregulated immune cells into two binary matrices (one for upregulated and one for downregulated cells), and (c) identifying somatic mutation-driven immune cells by comparing the gene mutation status with each immune cell in the binary matrices across all samples.

### Inference of the Relative Abundance Matrix of Immune Cells

Recently, various computational approaches have been developed for quantifying tumor-infiltrating immune cells from gene expression data of human bulk tumors. The SMDIC package provides three methods for estimating the relative infiltration abundance of different cell types in the tumor microenvironment (TME), namely, xCell (Aran et al., 2017), ssGSEA estimated method using the signatures of (Bindea et al., 2013; Senbabaoglu et al., 2016), and CIBERSORT (Newman et al., 2015). Through these methods, the relative abundance matrix of immune cells is inferred with cells as rows and samples as columns (Figure 1A). In the package, the default method is xCell, which estimates abundances of 64 cell types including adaptive and innate immunity cells, hematopoietic progenitors, epithelial cells, and extracellular matrix cells (Aran et al., 2017). The users can choose the method they are interested in.

### Detection of Differential Abundance Immune Cells With Respect to the Gene Mutation Status

The somatic mutation data used in the package are the mutation annotation file (MAF) format. We extract the non-silent somatic mutations (non-sense mutation, missense mutation, frame-shift indels, splice site, non-stop mutation, translation start site, inframe indels) in protein-coding regions and built a binary mutations matrix, in which 1 represents any mutation occurs in a particular gene in a particular sample; otherwise, the element is 0. The genes with a particular mutation frequency greater than a given threshold value are retained for the following analysis. Then, for a given mutation gene, the significance analysis of microarrays (SAM) method (Tusher et al., 2001) is used to detect differential abundance immune cells with respect to the mutation status (defined by binary mutation vector) across all the samples. With a false discovery rate (FDR) < 0.05 in SAM, the immune cells were deemed as significantly dysregulated between mutation and non-mutation samples and were considered to be correlated with the gene mutation status. Then, the abundance matrix elements of significantly dysregulated immune cells are converted to *z*-scores by row. For the upregulated (or downregulated) immune cells, any element with a *z*-score > 2.0 (or ≤ 2.0) is assigned with 1; otherwise, the element is 0 (Figure 1B). Thus, we obtain two binary abundance matrices for upregulated and downregulated immune cells, respectively.

### Identification of Somatic Mutation-Driven Immune Cells

For a particular mutation gene, we compare the binary mutation vector with each binary cell abundance vector in the upregulated or downregulated immune cell–matrix, respectively, and a 2 × 2 contingency table is constructed. The Fisher’s exact test is applied to recover the cells that had drastic mutation-correlated upregulated or downregulated response (Figure 1C). This process is repeated for each immune cell in the binary abundance matrices. To correct for multiple comparisons, we adjust the exact test *p*-values by using the FDR method proposed by Benjamini and Hochberg (1995). The immune cells with the default FDR < 0.05 are deemed as statistically significant mutation-correlated and may be driven by the somatic mutation. We repeat the above process for each mutated gene.

To further test if the somatic mutation-specific immune cells are associated with the prognosis of tumor patients, we constructed a signature by using immune cells driven by a particular gene mutation. The immune cell risk score was calculated with a prognostic score model for each patient based on the abundances of cells. The model is as follows:

(1) $$R\,i\,s\,k\,s\,c\,o\,r\,e=\sum_{k\in S}\beta_{k}\,a_{k}$$

where *S* is the set of cells correlated with a particular gene mutation, *a*_*k*_ is the abundance of cell *k*, and *β_*k*_* is the regression coefficient of a multivariate Cox proportional hazard regression model estimated on *a*_*k*_ and the overall survival data. According to the median of risk scores of the immune cell signature, patients are classified into high-risk and low-risk groups.

## Results

We selected the GDC TCGA Breast Cancer (BRCA) data cohorts^1^ as examples to explain the application of the SMDIC package. We downloaded the MAF file (derived from VarScan 2) from the GDC data portal, which is obtained from TCGA whole-exome sequencing (WES) data. Meantime, the RNA-seq FPKM gene expression data and the associated clinical data were also downloaded. These data have been deposited in Supplementary Data.

### Identification of Immune Cells Driven by Somatic Mutations in Breast Cancer

For the expression data, log-transformed FPKM expression values were used and were inputted into the “exp2cell” function for inferring the relative abundance matrix of infiltrated immune cells. The “exp2cell” function provides three methods for inferring the cell abundance matrix, namely, xCell (Aran et al., 2017), ssGSEA estimated method using the immune signatures of Bindea et al.(2013; Senbabaoglu et al., 2016), and CIBERSORT (Newman et al., 2015). The users can use the argument “method” to select the method they are interested in. The default argument is “xCell” method. Thus, a cell abundance matrix with 64 cells and 974 samples was obtained.

We then extracted the non-silent somatic mutations (non-sense mutation, missense mutation, frame-shift indels, splice site, non-stop mutation, translation start site, inframe indels) in protein-coding regions from the MAF file and built a binary mutation matrix, in which 1 represents any mutation occurs in a particular gene in a particular sample; otherwise, the element is 0. The “maf2matrix” function was used to implement this process. Alternatively, the users can directly input a binary mutation matrix which may not be derived from the MAF file. This will increase the usability of our package. With a given mutation frequency threshold (the default value is 1%), a binary matrix with 821 mutations and 974 samples was obtained.

In the SMDIC package, the “mutcorcell” function is implemented to identify somatic mutation-specific immune cell response by inputting the abundance matrix of immune cells and binary mutations matrix. This function firstly detects differential abundance immune cells with respect to a particular gene mutation status with the SAM method (Tusher et al., 2001). The immune cells with the default FDR < 0.05 in SAM are deemed as significantly dysregulated between mutation and non-mutation samples and are considered to be correlated with the gene mutation status. The significantly dysregulated immune cells are extracted and their abundance matrix elements are converted to *z*-scores by row. Then, the *z*-score abundance matrix is converted to two binary abundance matrices for upregulated and downregulated immune cells, respectively. Specifically, for the upregulated (or downregulated) immune cells, any element with a *z*-score > 2.0 (or ≤ 2.0) is assigned with 1; otherwise, the element is 0 (Figure 1B). Finally, for a particular mutation gene, the binary mutation vector is compared with each binary cell abundance vector in the upregulated and downregulated cell matrices, respectively, and a 2 × 2 contingency table is constructed. Moreover, the Fisher’s exact test is applied to recover the cells that had drastic mutation-correlated upregulated or downregulated response. This process is repeated for each immune cell in the binary abundance matrices. To correct for multiple comparisons, the exact test *p*-values are adjusted by using the FDR method (Benjamini and Hochberg, 1995), and the immune cells with the default FDR < 0.05 are deemed as statistically significant mutation-correlated and may be driven by the somatic mutation. The above process is repeated for each mutated gene.

The “mutcorcell” function will output the summary results of somatic mutation-driven immune cells and the detailed information of immune cells for each mutation, which are used as input data for the visualization functions. The detailed commands are listed in the Supplementary Material, and the GDC TCGA breast cancer datasets (mutation data, gene expression data, and survival data of patients) are stored in Supplementary Data. The summary results of immune cells driven by somatic mutation genes are listed in Table 1, and many of them have been reported in the recent literature. For example, regulatory T cells (Treg) have been found to be associated with a high mutation rate of TP53 genes in breast cancer (Oshi et al., 2020). The TP53 mutant has been identified to be associated with a higher expression of cytotoxic T-cell lymphocytes, natural killer (NK) cells, and Th1 genes characteristic of a proinflammatory immune cell signature (Agupitan et al., 2020). E-cadherin (CDH1) has been reported to target platelet endothelial cell adhesion molecule-1 (PECAM-1) for ubiquitination and degradation in endothelial cells (Liu et al., 2020), whose mutation may regulate endothelial cell homeostasis. Our results are consistent with these findings, and SMDIC could also identify some additional immune cells driven by these mutations, which may provide some new biological insights.

**TABLE 1:** The summary results of immune cells driven by somatic mutation genes.

Gene	Cells	Cell count	Mutation rate
*TP53*	Astrocytes, CD8^+^ naive T cells, CD8^+^ Tem, DC, epithelial cells, keratinocytes, macrophages, macrophages M1, melanocytes, NK cells, pDC, pericytes, plasma cells, pro-B cells, sebocytes, Tgd cells, Th1 cells, Th2 cells, Tregs	19	0.34
*CDH1*	Adipocytes, CD4^+^ Tcm, chondrocytes, CMP, endothelial cells, fibroblasts, HSC, ly endothelial cells, megakaryocytes, mesangial cells, mv endothelial cells, NKT	12	0.13
*NBPF14*	CD8^+^ Tem, DC, iDC, keratinocytes, pro-B cells, sebocytes	6	0.013
*OBSCN*	Epithelial cells, keratinocytes, pro-B cells, sebocytes, Th1 cells	5	0.030
*NF1*	aDC, B cells, memory B cells, pDC, plasma cells	5	0.037
*ANKRD30A*	Keratinocytes, sebocytes, Th1 cells, Th2 cells	4	0.015
*ARAP3*	CD8^+^ naive T cells, NK cells, preadipocytes, Th1 cells	4	0.010
*NOS1*	CD4^+^ Tem, CD8^+^ T cells, CD8^+^ Tcm, pDC	4	0.012
*PCDH19*	CD4^+^ memory T cells, CD4^+^ T cells, CD8^+^ Tem, NK cells	4	0.018
*KCNA4*	CD8^+^ T cells, CD8^+^ Tcm, NK cells, pDC	4	0.011
*SCN5A*	CD8^+^ T cells, CD8^+^ Tcm, plasma cells	3	0.013
*ACTN2*	NK cells, pDC, Th2 cells	3	0.011
*FBXW7*	MEP, Th1 cells, Th2 cells	3	0.016
*TRPS1*	CD4^+^ Tem, MSC, skeletal muscle	3	0.011

**The mutation genes correlated at least three significant cells are shown.**

Moreover, most of these cells driven by somatic mutations have been proposed to be associated with the progress of breast cancer. For example, in the TP53 mutation, 18 cells were identified (Table 2). It was proposed that type 1 T-helper (Th1) cells eradicated tumor mass by inducing cellular immunity, and type 2 T-helper (Th2) cells destroyed the tumor by inducing tumor necrosis (Nishimura et al., 1999). Macrophages M1 cells generate interleukin (IL)-12 and tumor necrosis factor with antitumor effects in breast cancer cells (Jeong et al., 2019). NK cells were reported to be critical immune components in controlling breast tumor growth and dissemination (Liu et al., 2019). For the cells driven by CDH1, CD4^+^ central memory T cell (Tcm) was proposed to be negatively associated with recurrence-free survival in breast cancer (Deng et al., 2019). The above results indicate that the SMDIC method could effectively identify immune-related cells driven by somatic mutations and may help to facilitate the development of cancer immunotherapy.

**TABLE 2:** The detailed information of immune cells driven by the TP53 gene mutation.

Cell	Full name	*p*-value	FDR
Th2 cells	Type 2 T-helper cells	8.86E-13	4.16E-11
Sebocytes	Sebocytes	1.51E-09	3.55E-08
Macrophages M1	Macrophages M1	6.33E-09	9.91E-08
Astrocytes	Astrocytes	1.01E-07	1.19E-06
Keratinocytes	Keratinocytes	2.33E-07	2.02E-06
Th1 cells	Type 1 T-helper cells	2.58E-07	2.02E-06
Macrophages	Macrophages	0.00018	0.0012
Pericytes	Pericytes	0.00035	0.0020
Tregs	Regulatory T cells	0.00048	0.0025
NK cells	Natural killer cells	0.00054	0.0025
Epithelial cells	Epithelial cells	0.00086	0.0037
Pdc	Plasmacytoid dendritic cells	0.0020	0.0077
Tgd cells	Gamma delta T cells	0.0021	0.0077
Pro-B cells	Pro-B cells	0.0026	0.0089
CD8^+^ naive T cells	CD8^+^ naive T cells	0.0040	0.013
Melanocytes	Melanocytes	0.0052	0.015
DC	Dendritic cells	0.016	0.042
Plasma cells	Plasma cells	0.016	0.042
CD8^+^ Tem	CD8^+^ effector memory T cells	0.017	0.043

### Presentation of the Results

**FIGURE 2:**
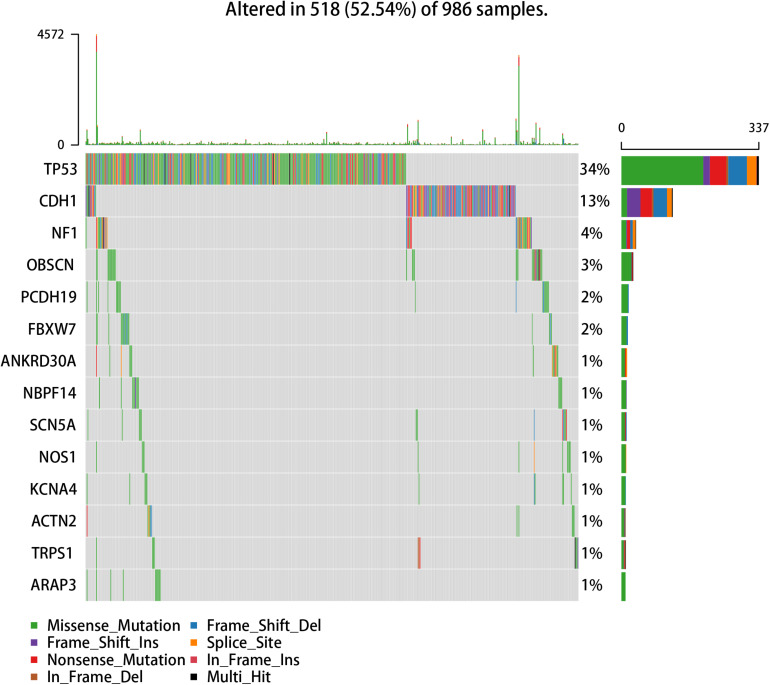
Waterfall plot of mutation genes that drive immune cells in the GDC TCGA breast cancer dataset.

**FIGURE 3:**
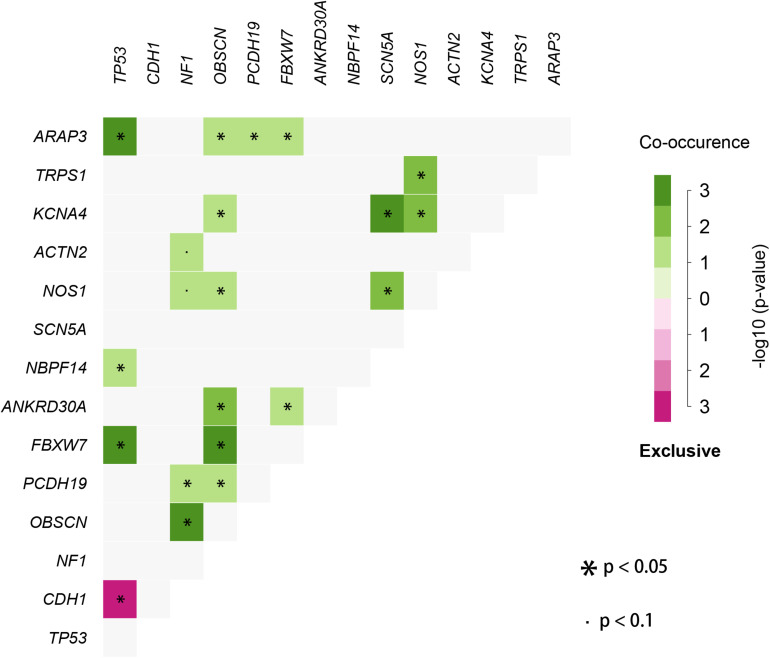
Co-occurrence and mutual exclusivity plots between mutation genes correlated with immune cells.

**FIGURE 4:**
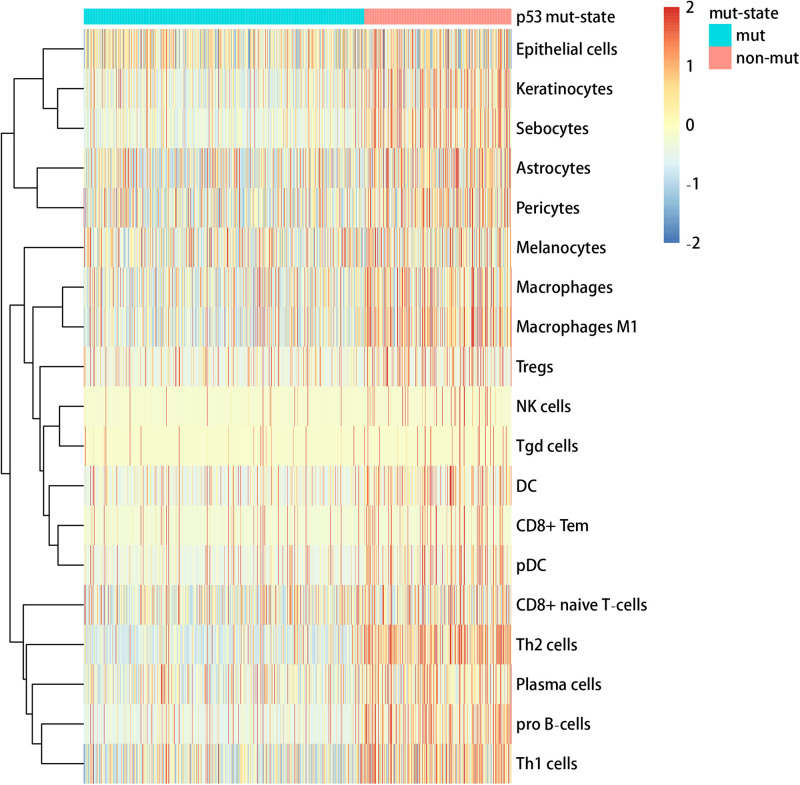
Heat map of cell abundance between TP53 mutation and non-mutation status.

**FIGURE 5:**
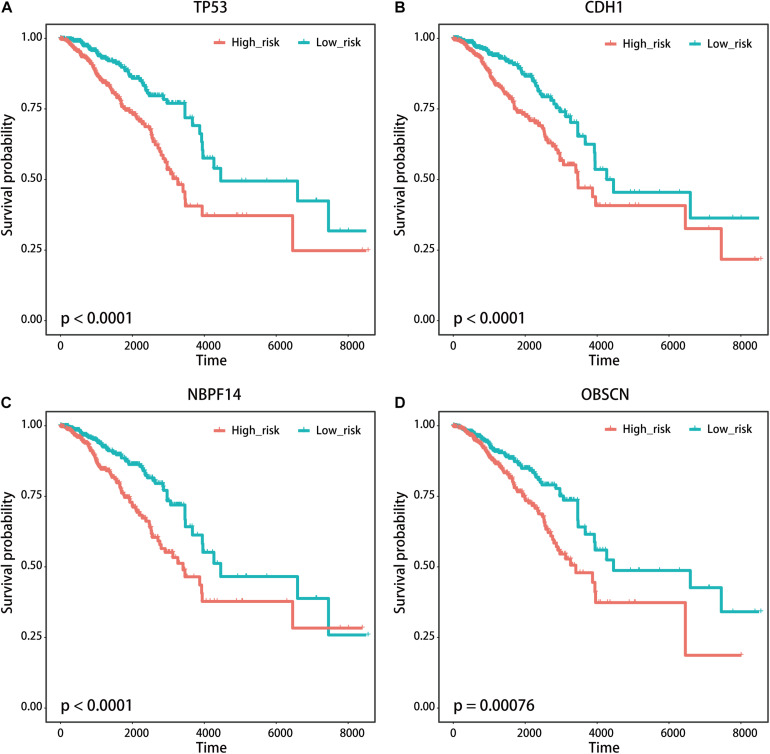
Kaplan–Meier survival curves of patients with breast cancer classified into high- and low-risk groups based on the immune cells driven by **(A)** TP53, **(B)** CDH1, **(C)** NBPF14, and **(D)** OBSCN mutations, respectively.

We used the visualization function of the SMDIC package to demonstrate the analysis results of breast cancer. The detailed commands for these visualization functions are listed in Supplementary Material. The “plotwaterfall” function is applied to show the waterfall plot of mutation genes that drive immune cells (Figure 2). To further analyze the correlation between these mutation genes, we used the “plotCoocMutex” function to plot the co-occurrence and mutual exclusivity plots (Figure 3). TP53 and CDH1 show significant mutual exclusions (p < 0.05). For a better display of the immune cell response triggered by somatic mutation, we used the “heatmapcell” function to plot a heat map to show the significant difference of cell abundance between gene mutation and non-mutation status. We selected the immune cells driven by TP53 as an example to show their heat map (Figure 4). We can see that the heat maps of immune cells display two obviously different blocks between TP53 mutation and non-mutation samples. From the heat maps of immune cells driven by CDH1, a similar result was obtained (Supplementary Figure 1). We then used the “survcell” function to plot the Kaplan–Meier survival curves of patients classified into high- and low-risk groups using the median of the immune cell risk score (see Implementation section). The immune cells driven by TP53, CDH1, NBPF14, and OBSCN mutations were, respectively, selected to calculate the immune risk score for plotting Kaplan–Meier survival curves (Figures 5A–D). It can be seen that immune cells driven by these mutations could be used as prognostic signatures. These results indicate that these mutations may be used as neoantigens for immunotherapy.

### Robustness Analysis of the SMDIC Method

We first tested if the immune cells driven by the specific somatic mutation had the same or similar prognostic value in different independent datasets. As the public cancer datasets with both genome and transcriptome are seldom, we used the mutation-specific immune cell signatures (see *Implementation* section) obtained from the TCGA breast cancer dataset to test their prognosis performance in independent gene expression datasets. We enrolled four independent datasets of breast cancer, namely, GSE1992 (Hu et al., 2006), GSE3143 (Bild et al., 2006), GSE1456 (Pawitan et al., 2005), and GSE7390 (Desmedt et al., 2007) datasets. The cell signature derived from TP53 mutation was firstly used to classify patients from four independent breast cancer expression datasets, respectively. For each independent dataset, the “exp2cell” was applied to convert gene expression profiles to cell abundance profiles, and the patients were divided into high-risk and low-risk groups based on the cell signature. Interestingly, the *p*-values of log-rank tests in these four datasets were all < 0.05 (Supplementary Figures 2A–D). For the cell signature driven by CDH1, it was shown that the CDH1-specific cell signature could classify patients into high- and low-risk groups in three of the above four datasets (log-rank tests *p*-value < 0.05, Supplementary Figures 3A–D). We also found that the cell signatures derived from NBPF14 and OBSCN mutations respectively obtained similar results (Supplementary Figures 4, 5). These results confirmed that the mutation-driven immune cells could be used as prognostic signatures.

**FIGURE 6:**
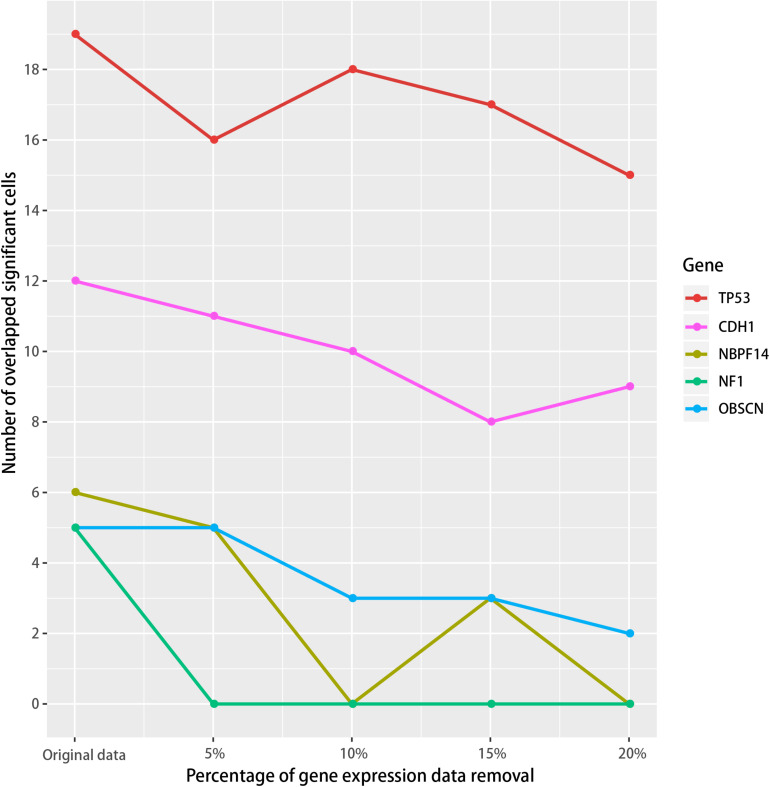
Robustness analysis of the SMDIC method. The gene expression values were randomly removed from 5 to 20% at 5% intervals. For each removal, the overlapped number of the immune cells with the original results was calculated for the mutation genes which activate at least five immune cells.

We then tested if the SMDIC method is robust to missing data in the gene expression profiles. To do this, we performed data removal tests using the GDC TCGA breast cancer expression profiles. For the gene expression data, we randomly removed the genes in the original expression profiles from 5 to 20% at 5% intervals. For each data removal, we repeated the SMDIC method to identify somatic mutation-specific immune cells. We then compared these results with our original results. The mutation genes which trigger at least five immune cells, namely, TP53, CDH1, OBSCN, NF1 and NBPF14, were used to test how many significant immune cells are overlapped. With the data removal increasing, we found that the number of overlapped cells driven by each mutation gene fell slowly compared with the original data (Figure 6). Moreover, 89% (17/19), 66% (8/12), 60% (3/5), and 50% (3/6) cells were shared with our original results for the TP53, CDH1, NBPF14, and OBSCN mutations, respectively, even after removal of up to 15% of the gene expression data. These results indicate that the SMDIC method is robust to missing data in the gene expression profiles.

We further tested if the SMDIC method is robust to different methods for estimating abundance of immune cell infiltration. The default method for estimating cell abundance is xCell (Aran et al., 2017) in the package. We used an alternative ssGSEA estimated method (Senbabaoglu et al., 2016) to test if the SMDIC method could obtain consistent results. In the GDC TCGA breast cancer datasets, the SMDIC method identified 19 and 15 TP53 mutation-specific immune cell response in the xCell and ssGSEA cell abundance estimated methods (Table 2 and Supplementary Table 1). There are only five overlapped immune cells, namely, type 1 T-helper (Th1) cell, type 2 T-helper (Th2) cell, gamma delta T cells (Tgd), regulatory T cells (Tregs), and NK cell. This is because xCell and ssGSEA use different cell estimated strategies and quantify different cell types. However, through calculating the Pearson correlation between xCell and ssGSEA values for relevant cell types driven by TP53 mutation, we observed relatively stronger levels of concordance (e.g., CD8^+^ naive T cells vs. CD8 T cells, Pearson *r* = 0.31, *p*-value < 1e-20; dendritic cells vs. activated dendritic cells, Pearson *r* = 0.53, *p*-value < 1e-20) (Supplementary Table 2). These validation results show that our somatic mutation-driven immune cell method is robust to different cell abundance estimated methods.

To further illustrate the applicability of our SMDIC method, we applied it to TCGA skin cutaneous melanoma (SKCM) data, which were downloaded from the GDC data portal (see text footnote 2). We inputted all the related data into the SMDIC package and performed the same operations like in the breast cancer data. The detailed results are listed in Supplementary Table 3. We found that some mutation cell associations identified by the method have been reported in the literature. For example, it has been proposed that deficiency of E3 ubiquitin ligase (ITCH) altered CD4 T-cell and B-cell responses in mice and humans (Field et al., 2020); HIVEP2 was found to play a crucial role in the control of Th2 cell differentiation by regulating NF-κB function, whose ortholog gene HIVEP3 may substitute for the function of HIVEP2 (Kimura et al., 2005). This indicates that the SMDIC method could identify mutation-specific cells in different cancers.

## Discussion

Recently, a number of calculated methods have been developed to identify dysregulated genes and pathways in cancer using molecular omics data (genome or transcriptome) (Cheng et al., 2019; Han et al., 2020; Sheng et al., 2021). Moreover, some methods were proposed to prioritize cancer candidate drugs and prognostic markers (Han et al., 2018, 2021; Di et al., 2019). However, the methods for identifying immune cells driven by specific somatic mutations are mainly based on biological experiments, and as far as we have known, no calculated tools could systematically identify mutation-specific immune cells.

In the study, SMDIC R package is an automated, computationally fast, and efficient tool for exploring somatic mutation-driven immune cell response by integrating genome and transcriptome data. The users input the somatic mutation data and gene expression data with the same samples; the package will return the immune cells triggered by each mutation and provide visualization of results. Moreover, our package is not specifically designed for breast cancers and can be generally applicable for various cancer datasets with genomic and transcriptome data. The SMDIC tool was designed to identify somatic mutation-specific immune cells by integrating high-throughput genomic and transcriptome data, which may help to find neoantigens and facilitate the development of personalized immunotherapy to patients with cancers.

## Data Availability Statement

The datasets presented in this study can be found in online repositories. The names of the repository/repositories and accession number(s) can be found in the article/Supplementary Material.

## Ethics Statement

Ethical review and approval was not required for the study on human participants in accordance with the local legislation and institutional requirements. Written informed consent for participation was not required for this study in accordance with the national legislation and the institutional requirements.

## Author Contributions

JH and YJ conceived and designed the study. BZ developed software. YY and XL analyzed the data and implemented the methodology. YJ revised the manuscript. JH drafted the manuscript. All authors read and agreed to the final version of the manuscript.

## Conflict of Interest

The authors declare that the research was conducted in the absence of any commercial or financial relationships that could be construed as a potential conflict of interest.

## Footnotes

1 https://portal.gdc.cancer.gov/
