# Supplementary material for: Identification of Somatic Mutation-Driven Immune Cells by Integrating Genomic and Transcriptome Data
Source: Front Cell Dev Biol. 2021 Jul 21;9:715275. doi: 10.3389/fcell.2021.715275 (PMC8335569; doi:10.3389/fcell.2021.715275)
Supplement: Supplementary file 1 [file Data_Sheet_1.doc]

**Supplementary Materials**

**Application of the SMDIC package**

The "mutcorcell" function will output the summary results of somatic mutations driven [immune](../../../../C:%5CProgram%20Files%20(x86)%5CYoudao%5CDict%5C7.5.2.0%5Cresultui%5Cdict%5C%3Fkeyword=immune)[cell](../../../../C:%5CProgram%20Files%20(x86)%5CYoudao%5CDict%5C7.5.2.0%5Cresultui%5Cdict%5C%3Fkeyword=cell)s and the detailed information of [immune](../../../../C:%5CProgram%20Files%20(x86)%5CYoudao%5CDict%5C7.5.2.0%5Cresultui%5Cdict%5C%3Fkeyword=immune)[cell](../../../../C:%5CProgram%20Files%20(x86)%5CYoudao%5CDict%5C7.5.2.0%5Cresultui%5Cdict%5C%3Fkeyword=cell)s for each mutation, which are used as input data for the visualization functions. The summary results of [immune](../../../../C:%5CProgram%20Files%20(x86)%5CYoudao%5CDict%5C7.5.2.0%5Cresultui%5Cdict%5C%3Fkeyword=immune)[cell](../../../../C:%5CProgram%20Files%20(x86)%5CYoudao%5CDict%5C7.5.2.0%5Cresultui%5Cdict%5C%3Fkeyword=cell)s driven by somatic mutation genes are listed in Table 1, and the detailed information of [immune](../../../../C:%5CProgram%20Files%20(x86)%5CYoudao%5CDict%5C7.5.2.0%5Cresultui%5Cdict%5C%3Fkeyword=immune)[cell](../../../../C:%5CProgram%20Files%20(x86)%5CYoudao%5CDict%5C7.5.2.0%5Cresultui%5Cdict%5C%3Fkeyword=cell)s driven by TP53 mutation is shown in Table 2. The following commands can execute the SMDIC method.

*# load the SMDIC package.*

*>library (SMDIC)*

*# set the working directory to dir and load breast cancer gene expression data (TCGA.BRCA.ExpressionData.txt), survival data of patients (TCGA.BRCA.SurvivalData.txt), and mutation data of MAF file (TCGA.BRCA.SomaticData.maf). These datasets are stored in Supplementary Data.*

*>dir=".../BRCAdata/"*

*>setwd(dir)*

*>* *expressiondata<-read.table("TCGA.BRCA.ExpressionData.txt", header = TRUE, stringsAsFactors=FALSE, row.names=1)*

*>survivaldata<-read.table("TCGA.BRCA.SurvivalData.txt", header=TRUE, stringsAsFactors=FALSE)*

*# convert MAF format data to a binary mutations matrix with "maf2matrix" function. The argument "percent" is the mutation frequency in a patient cohort.*

*>mutmatrix<-maf2matrix(maffile="TCGA.BRCA.SomaticData.maf", percent=0.01,* *nonsynonymous=TRUE)*

*# convert expression profiles to cell abundance profiles with "exp2cell " function.*

*>cellmatrix<-exp2cell(exp=expressiondata, method="xCell")*

*# perform the SMDIC method. The argument "samfdr.cutoff" is the FDR threshold of the SAM method. The argument "nperms" is the times of permutation in SAM, and its default value is set at 100 ("nperms=100") in the package. To obtain a more stable result, "nperms" is better set to 1000. The argument "fisher.cutoff" is the threshold of Fisher's exact test, and its default value is 0.05. If the argument "fisher.cutoff " is "T", the "fisher.cutoff " is applied to the FDR* *of the exact test, otherwise, the p-value of the exact test is used. The mutation genes which drive at least one cell are retained. The binary mutations matrix inputted into the function could be obtained from different data formats not limited to the MAF file derived from varscan2.*

*>mutcell<-mutcorcell (cellmatrix=cellmatrix, mutmatrix=mutmatrix, samfdr.cutoff=0.05, nperms =1000, fisher.cutoff=0.05, fisher.adjust=T)*

*# show the summary results of* [*immune*](../../../../C:%5CProgram%20Files%20(x86)%5CYoudao%5CDict%5C7.5.2.0%5Cresultui%5Cdict%5C%3Fkeyword=immune)[*cell*](../../../../C:%5CProgram%20Files%20(x86)%5CYoudao%5CDict%5C7.5.2.0%5Cresultui%5Cdict%5C%3Fkeyword=cell)*s driven by somatic mutations.*

*>summary<-mutcellsummary(mutcell=mutcell, mutmatrix=mutmatrix, cellmatrix=cellmatrix)*

*# show the detailed information of* [*immune*](../../../../C:%5CProgram%20Files%20(x86)%5CYoudao%5CDict%5C7.5.2.0%5Cresultui%5Cdict%5C%3Fkeyword=immune)[*cell*](../../../../C:%5CProgram%20Files%20(x86)%5CYoudao%5CDict%5C7.5.2.0%5Cresultui%5Cdict%5C%3Fkeyword=cell)*s driven by TP53 mutation.*

*>gene2cellsummary <-gene2cellsummary (gene="TP53", mutcell=mutcell)*

**Visualization of results**

For the analysis results of breast cancer, we used the visualization function of the SMDIC package to demonstrate. The "plotwaterfall" function is applied to show the waterfall plot of mutation genes that drive immune cells (Figure 2). To further analyze the correlation between these mutation genes, we used the "plotCoocMutex" function to plot the co-occurrence and mutual exclusivity plots (Figure 3). TP53 and CDH1 show significant mutual exclusions (p<0.05). The commands and results are listed as follows.

*# plot the waterfall of mutation genes that drive immune cells. The argument "cellnumcuoff " is the threshold of immune cells, and the mutation genes which regulated immune cells at equal or greater than this threshold (the default value is 3) are shown in the waterfall plot.*

*> plotwaterfall (maffile="TCGA.BRCA.SomaticData.maf", mutcell.summary=summary, cellnumcuoff=3)*

*# plot the co-occurrence and mutual exclusivity plots between mutation genes.*

*> plotCoocMutex (maffile="TCGA.BRCA.SomaticData.maf", mutcell.summary=summary, cellnumcuoff=3)*

For better display of the im*mune cell responses triggered by som*atic mutation, we used the "heatmapcell" function to plot a heat map to show the significant difference of cell abundance between gene mutation and non-mutation status. We selected the immune cells driven by TP53 as an example (Figure 4). The commands and results are shown as follows.

*# plot heatmaps for the cells driven by TP53 mutation.*

*>**heatmapcell (gene="TP53", mutcell=mutcell, cellmatrix=cellmatrix, mutmatrix=mutmatrix)*

We used the "survcell" function to show the Kaplan-Meier survival curves of patients classified into high- and low-risk groups using the median of the immune risk score (see "Implementation" in the manuscript). The immune cells driven by TP53 mutation were selected as an example to calculate the immune risk score for plotting Kaplan-Meier survival curves (Figure 5). The commands and results are shown as follows.

*# plot Kaplan-Meier survival curves based on immune cells driven by TP53 mutation.*

*> survcell (gene="TP53", mutcell=mutcell, cellmatrix=cellmatrix, surv=survivaldata, method="Multivariate")*

**
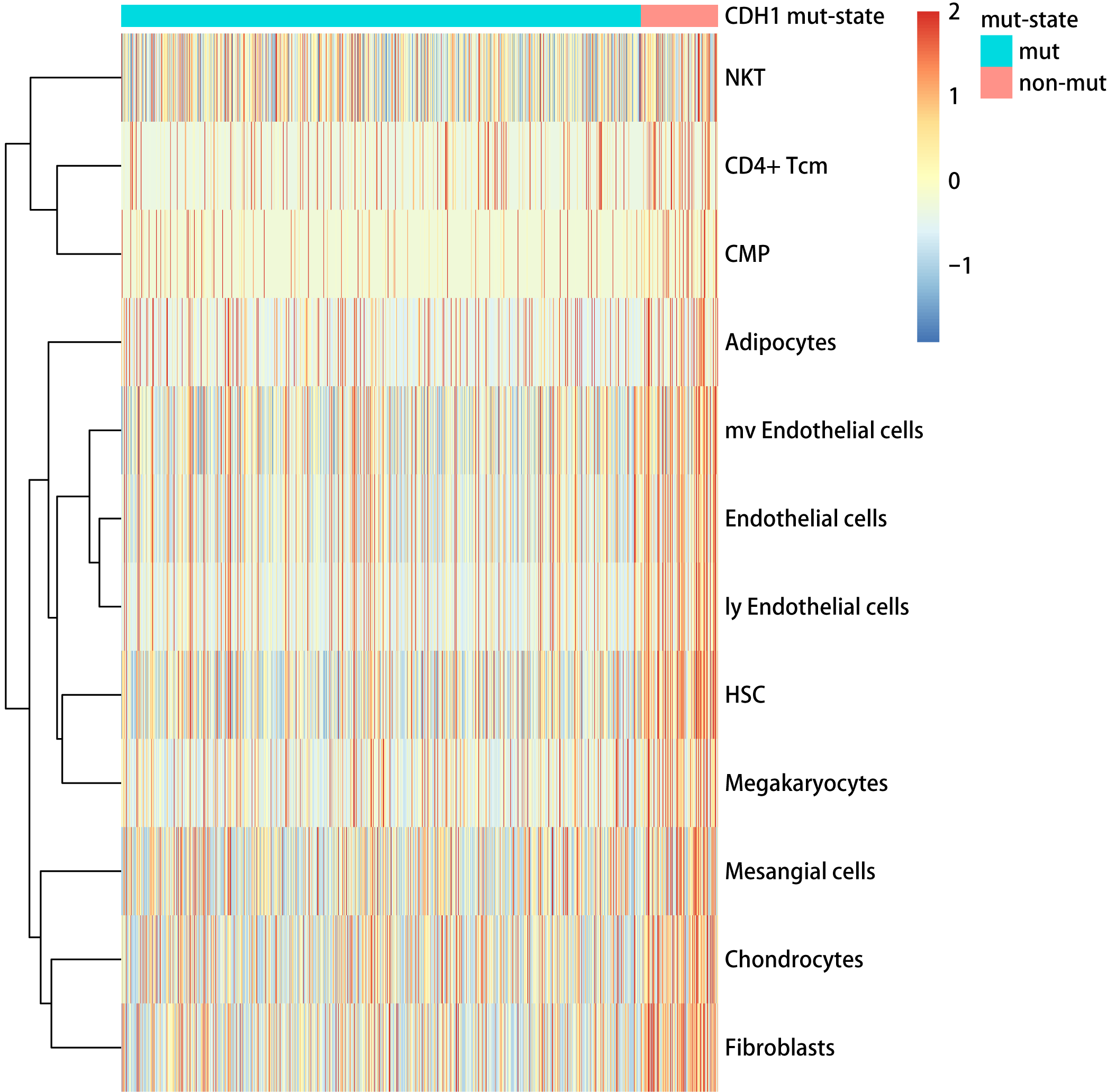
**

Figure S1. Heat map of cell abundance between CDH1 mutation and non-mutation status.


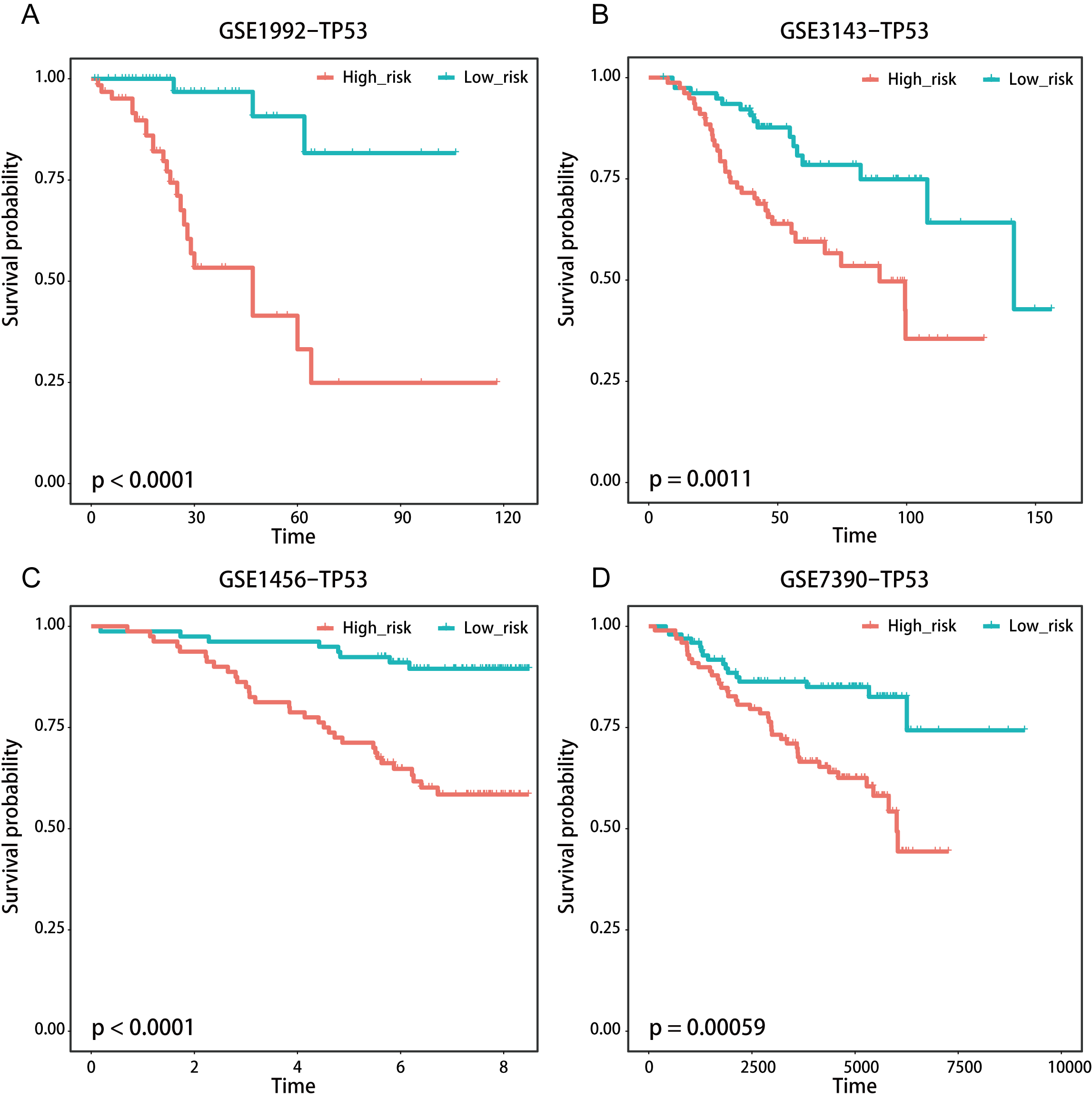


Figure S2. Kaplan-Meier survival curves of patients classified into high- and low-risk groups using the TP53-specific [cell](../../../../C:%5CProgram%20Files%20(x86)%5CYoudao%5CDict%5C7.5.2.0%5Cresultui%5Cdict%5C%3Fkeyword=cell) signature in the (A) GSE1992, (B)GSE3143, (C) GSE1456, and (D) GSE7390.

**
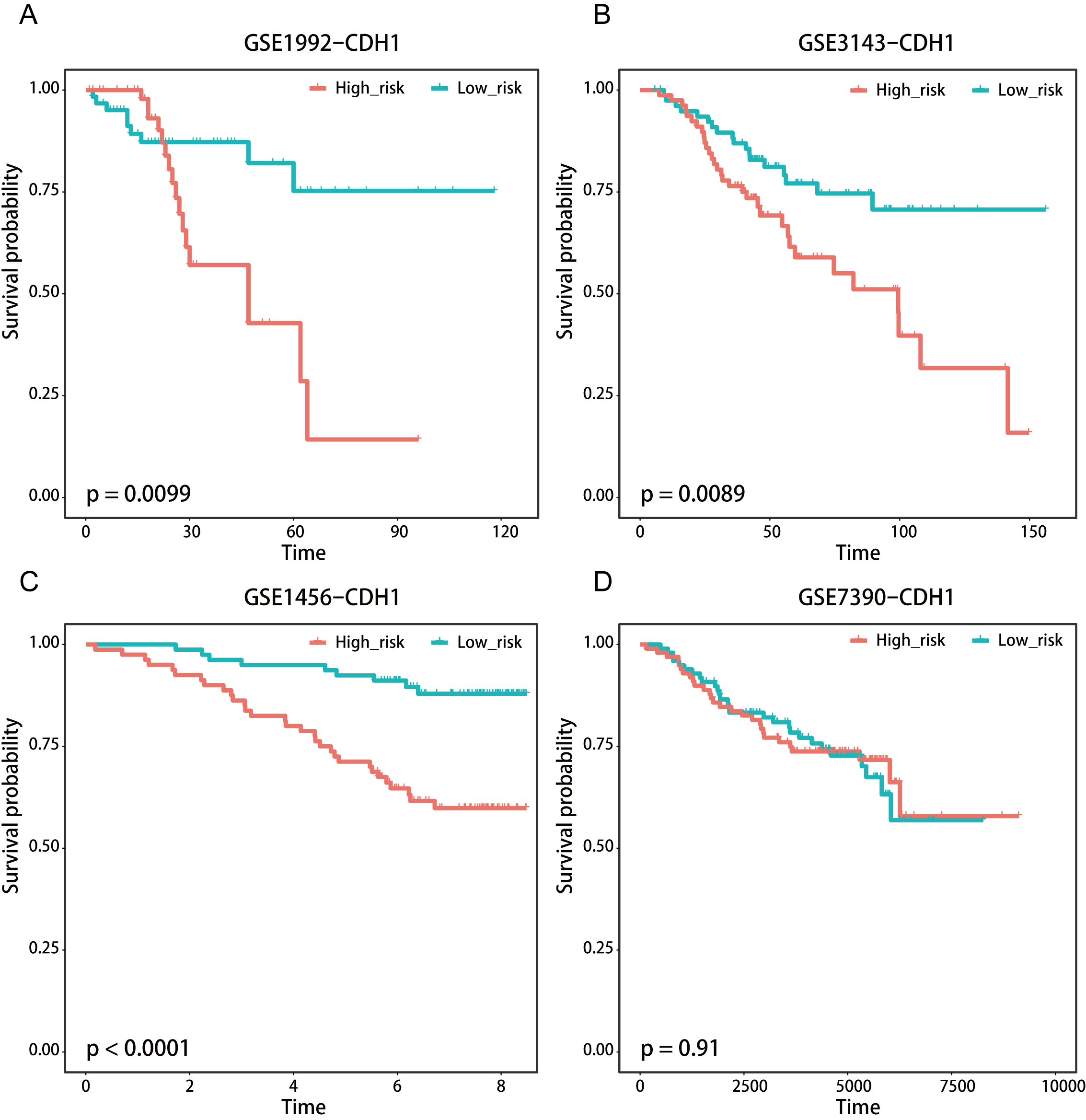
**

Figure S3. Kaplan-Meier survival curves of patients classified into high- and low-risk groups using the CDH1-specific [cell](../../../../C:%5CProgram%20Files%20(x86)%5CYoudao%5CDict%5C7.5.2.0%5Cresultui%5Cdict%5C%3Fkeyword=cell) signature in the (A) GSE1992, (B)GSE3143, (C) GSE1456, and (D) GSE7390.

**
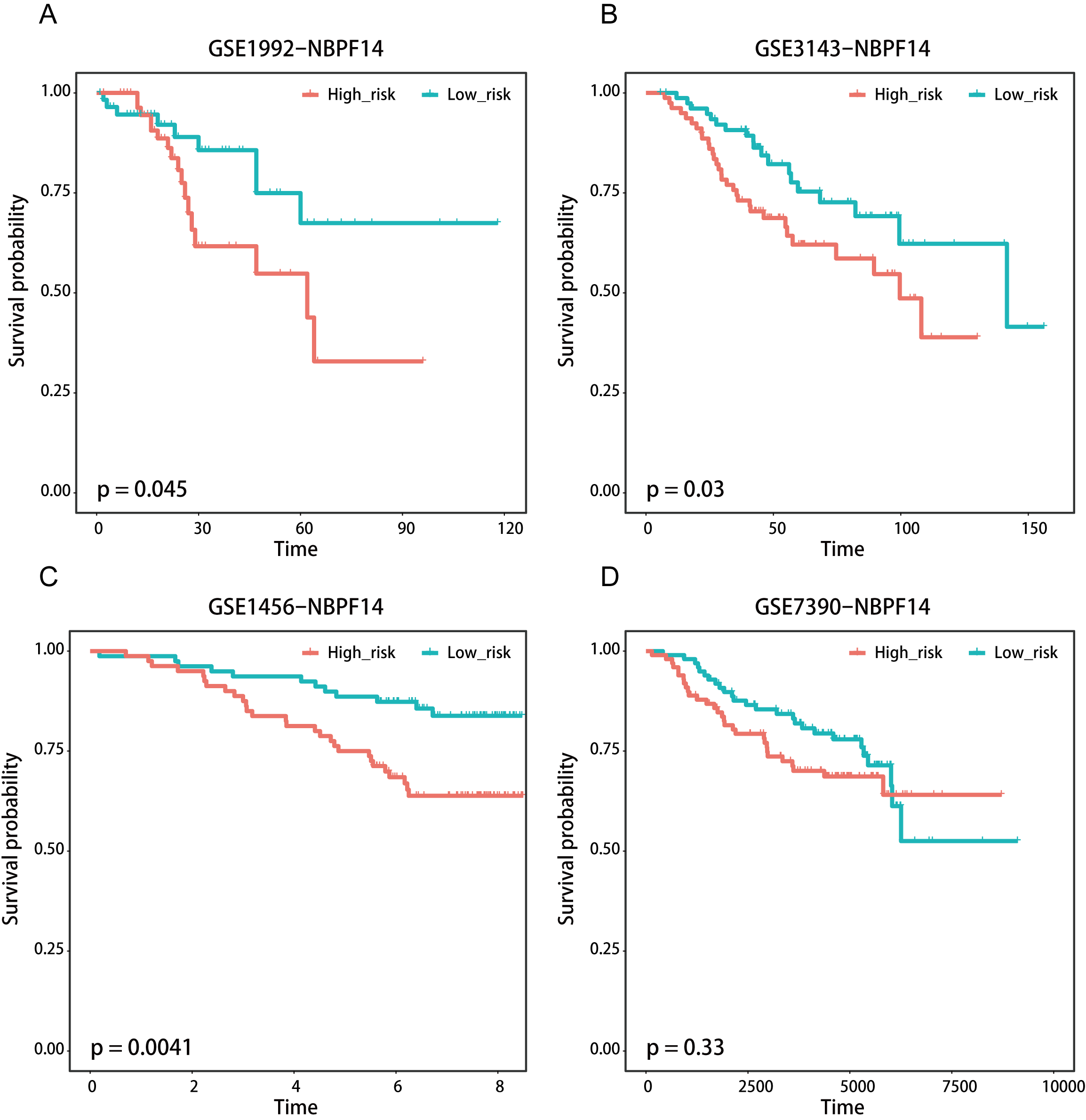
**

Figure S4. Kaplan-Meier survival curves of patients classified into high- and low-risk groups using the NBPF14-specific [cell](../../../../C:%5CProgram%20Files%20(x86)%5CYoudao%5CDict%5C7.5.2.0%5Cresultui%5Cdict%5C%3Fkeyword=cell) signature in the (A) GSE1992, (B)GSE3143, (C) GSE1456, and (D) GSE7390.


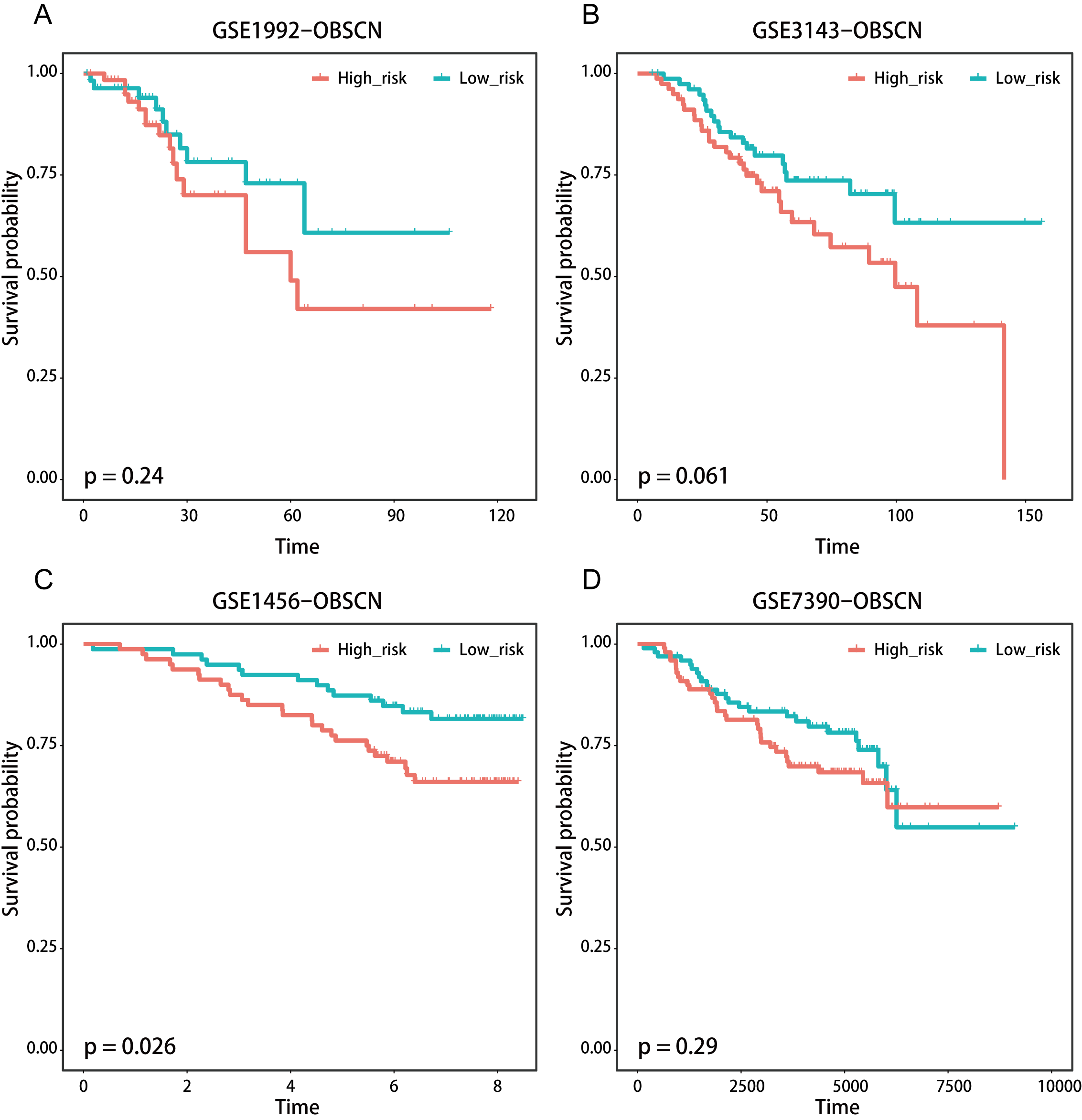


Figure S5. Kaplan-Meier survival curves of patients classified into high- and low-risk groups using the OBSCN -specific [cell](../../../../C:%5CProgram%20Files%20(x86)%5CYoudao%5CDict%5C7.5.2.0%5Cresultui%5Cdict%5C%3Fkeyword=cell) signature in the (A) GSE1992, (B) GSE3143, (C) GSE1456, and (D) GSE7390.

**Table S1.** The detailed information of [immune](../../../../C:%5CProgram%20Files%20(x86)%5CYoudao%5CDict%5C7.5.2.0%5Cresultui%5Cdict%5C%3Fkeyword=immune)[cell](../../../../C:%5CProgram%20Files%20(x86)%5CYoudao%5CDict%5C7.5.2.0%5Cresultui%5Cdict%5C%3Fkeyword=cell)s (estimated by ssGSEA) driven by the TP53 gene mutation.

| Cell | Full name | pvalue | fdr |
| --- | --- | --- | --- |
| Eosinophils | Eosinophils | 1.44E-10 | 3.45E-09 |
| NK cells | Natural killer cells | 1.94E-08 | 2.33E-07 |
| Th17 cells | Type 17 T-helper cells | 4.34E-08 | 3.47E-07 |
| NK CD56bright cells | NK CD56bright cells | 1.33E-05 | 8.01E-05 |
| iDC | Immature DCs | 4.03E-05 | 0.00019 |
| Mast cells | Mast cells | 0.00010 | 0.00036 |
| Tgd cells | Gamma delta T-cells | 0.00010 | 0.00036 |
| Th1 cells | Type 1 T-helper cells | 0.0002 | 0.00074 |
| aDC | Activated DCs | 0.0011 | 0.0031 |
| Tem cells | Effect memory T cells | 0.0048 | 0.010 |
| Treg cells | regulatory T cells | 0.0045 | 0.010 |
| CD8 T cells | CD8 T cells | 0.0097 | 0.018 |
| Tfh cells | T follicular helper cells | 0.0098 | 0.018 |
| Tcm cells | Centermemory T cells | 0.016 | 0.027 |
| Th2 cells | Type 2 T-helper cells | 0.021 | 0.033 |

**Table S2.** Correlation between Xcell and ssGSEA immune scores. Pearson correlation (and p-values in parentheses) between Xcell scores (red text) and ssGSEA scores obtained with Bindea et al immune signatures (blue text).

|  |  | ssGSEA cell estimated method | | | | |
| --- | --- | --- | --- | --- | --- | --- |
| Xcell method |  | CD8 T cells | |  |  | |
|  | CD8+ naive T−cells | 0.31 (<1e-20) | |  |  | |
|  |  |  |  |  |  |  |
|  |  | CD8 T cells | |  |  | |
|  | CD8+ effector memory T−cells | 0.43 (<1e-20) | |  |  | |
|  |  |  |  |  |  |  |
|  |  | Activated Dendritic Cells | | Immature Dendritic Cells |  | |
|  | Dendritic Cells | 0.53 (<1e-20) | | 0.56 (<1e-20) |  | |
|  |  |  |  |  |  |  |
|  |  | Activated Dendritic Cells | | Immature Dendritic Cells |  | |
|  | Plasmacytoid Dendritic Cells | 0.59 (<1e-20) | | 0.22 (2.66E-15) |  | |
|  |  |  |  |  |  |  |
|  |  | | Center memory T cells | Effect memory T cells | T follicular helper cells | |
|  | Gamma delta T−cells | | 0.22 (1.62E-14) | 0.20 (3.79E-12) | 0.21 (4.66E-14) | |

**Table S3.** The summary results of [immune](../../../../C:%5CProgram%20Files%20(x86)%5CYoudao%5CDict%5C7.5.2.0%5Cresultui%5Cdict%5C%3Fkeyword=immune)[cell](../../../../C:%5CProgram%20Files%20(x86)%5CYoudao%5CDict%5C7.5.2.0%5Cresultui%5Cdict%5C%3Fkeyword=cell)s driven by somatic mutation genes for TCGA Skin Cutaneous Melanoma (SKCM) data. The top 10 mutation genes that regulate the most immune cells were shown

| Gene | Cells | Cell count | Mutation rate |
| --- | --- | --- | --- |
| ITCH | B-cells,CD4+ memory T-cells,CD4+ naive T-cells,CD4+ T-cells, Class-switched memory B-cells, CLP, Memory B-cells, naive B-cells, pro B-cells | 9 | 0.017 |
| RBBP8NL | CD8+ T-cells,CD8+ Tcm,CD8+ Tem, CLP, Myocytes, Skeletal muscle,Th2 cells | 7 | 0.026 |
| SEH1L | B-cells,CD4+ naive T-cells,CD4+ T-cells, Memory B-cells, naive B-cells, pDC, Platelets | 7 | 0.011 |
| SLC9A6 | ly Endothelial cells, Macrophages M2,MSC,mv Endothelial cells, NKT, Smooth muscle,Th1 cells | 7 | 0.01 |
| HIVEP3 | Basophils, Melanocytes, pro B-cells, Skeletal muscle, Tgd cells,Th2 cells | 6 | 0.11 |
| CLASP1 | B-cells,CD4+ memory T-cells,CD4+ naive T-cells,CD4+ T-cells, cDC, naive B-cells | 6 | 0.026 |
| PLK4 | CD4+ naive T-cells, Class-switched memory B-cells, Endothelial cells, ly Endothelial cells, Megakaryocytes, mv Endothelial cells | 6 | 0.011 |
| CUEDC2 | Astrocytes, Chondrocytes, Fibroblasts, Myocytes, Skeletal muscle,Th2 cells | 6 | 0.013 |
| BPIFC | CD4+ memory T-cells,CD4+ T-cells, Class-switched memory B-cells, Memory B-cells, naive B-cells, pro B-cells | 6 | 0.030 |
| GRHL3 | CD4+ memory T-cells, CLP, Mast cells, Plasma cells, Tregs | 5 | 0.013 |
