# Supplementary material for: Identification of Somatic Mutation-Driven Immune Cells by Integrating Genomic and Transcriptome Data
Source: Front Cell Dev Biol. 2021 Jul 21;9:715275. doi: 10.3389/fcell.2021.715275 (PMC8335569; doi:10.3389/fcell.2021.715275)
Supplement: Supplementary file 2 [file Data_Sheet_2.zip › Supplementary Data.DOCX]

Link to raw data:

[https://www.jianguoyun.com/p/DelTQ3wQ9tjGCRiTnfgD](https://www.jianguoyun.com/p/Df735skQ9tjGCRjamPgD%22.)
